# Effects of multiple vitamin E levels and two fat sources in diets for swine fed to heavy slaughter weight of 150 kg: I. Growth performance, lean growth, organ size, carcass characteristics, primal cuts, and pork quality

**DOI:** 10.1093/tas/txad086

**Published:** 2023-07-28

**Authors:** Ding Wang, Young Dal Jang, Marlee Kelley, Gregg K Rentfrow, Michael J Azain, Merlin D Lindemann

**Affiliations:** Department of Animal and Food Sciences, University of Kentucky, Lexington, KY 40546, USA; Department of Animal and Dairy Science, University of Georgia, Athens, GA 30602, USA; Department of Animal and Food Sciences, University of Kentucky, Lexington, KY 40546, USA; Department of Animal and Food Sciences, University of Kentucky, Lexington, KY 40546, USA; Department of Animal and Dairy Science, University of Georgia, Athens, GA 30602, USA; Department of Animal and Food Sciences, University of Kentucky, Lexington, KY 40546, USA

**Keywords:** fat, vitamin E level, heavy slaughter weight pigs, growth, meat quality

## Abstract

The study objective was to evaluate the effect of two fat source and graded levels of vitamin E (**VE**) supplementation on growth performance, carcass characteristics, and meat quality of pigs at heavy slaughter weight (150 kg). A total of 48 individually-fed pigs (24 barrows, 24 gilts; 28.44  ± 2.69 kg) were blocked by sex and weight and randomly assigned to eight dietary treatments in a 2 × 4 factorial arrangement. Fat treatments were 5% tallow (**TW**) and distiller’s corn-oil (**DCO**) in the diets. The VE treatments included four levels of α-tocopheryl-acetate (11, 40, 100, and 200 ppm). Growth performance, carcass traits, organ weight, primal cuts, and pork quality were measured. Increasing dietary VE supplementation levels linearly increased overall Average daily gain (ADG) and average daily feed intake (*P *< 0.05), with an interaction between fat sources and VE supplementation levels on cumulative ADG (*P *< 0.05) during phases 1 and 3 (28 to 100 kg) and 1 to 4 (28 to125 kg) wherein ADG in the pigs fed the DCO diet, but not the TW diet, increased with increasing dietary VE supplementation level. A similar interaction was observed in 24 h pH and picnic shoulder (*P* < 0.05). No notable effect of fat source was observed in growth performance. With increasing dietary VE supplementation levels, there were quadratic responses in pork pH at 45 min and 24 h postmortem with the highest value in 40 and 100 ppm of VE levels while TBARS values on day 7 postmortem decreased linearly (*P* < 0.05). Compared with the TW diet, the DCO diet resulted in greater TBARS values during 7 postmortem (*P* < 0.05; day 5, *P* = 0.09). These results demonstrated that increasing dietary VE supplementation level could enhance growth rate and feed intake and reduce lipid peroxidation of pork whereas the diet containing DCO as a fat source could negatively affect pork shelf-life and carcass characteristics and that increasing VE supplementation level had no notable interaction with fat sources for carcass characteristics.

## Teaser

1.Increasing vitamin E supplementation increases daily feed intake and resultant daily gain in the latter stages of growth and improves pork shelf life stability.

2.Corn oil, compared to tallow, generally did not affect growth but it did reduce pork quality.

## Introduction

Vitamin E (**VE**) is a strong anti-oxidant that protects both the live body and pork products from lipid peroxidation (Lauridsen et al., 2013; Ng et al., 2016) resulting in improved health, immunity, and meat quality in pigs (Guo et al., 2006; Niculita et al., 2007). Dietary VE can be deposited into different tissues but mainly stored in adipose tissues in the body (Schmölz et al., 2016). However, the dietary VE requirement estimate in NRC (2012) may be low if the desired response is to obtain a significant deposition of VE in tissues and improved shelf-life of pork.

Supplemental dietary fat sources increase energy density of diets and affect voluntary feed intake, feed efficiency, growth rate, and fatty acid (FA) composition and VE accumulation in pigs (Duran-Montgé et al., 2009; Liu et al., 2018; Silva-Guillen et al., 2020; Wang et al., 2022a). In our previous studies with only two supplementation levels of VE (11 and 200 IU/kg) fed to pigs slaughtered at heavier weight (150 kg), Wang et al. (2022a, 2022b) reported that: 1) addition of 5% fat in the diet reduced feed intake and increased feed efficiency, 2) different fat sources affected the FA composition in adipose tissues (backfat and belly fat), 3) fat sources interacted with VE supplementation on plasma VE concentrations and anti-oxidant capacity, and 4) there were minimal effects of VE supplementation and fat source observed on carcass traits and pork quality. Although there is a potential interaction between dietary fat source and VE in pigs because of the difference in the degree of saturation of fat sources affecting the VE absorption (Prévéraud et al., 2014; Wang et al. 2022a, 2022b), the most appropriate supplementation level of VE to improve feed efficiency and potentially affect carcass traits and pork quality when the degree of saturation of the diets has an extreme difference by using different fat sources such as tallow (**TW**) and corn oil has not been adequately evaluated.

Therefore, the objective of the current study was to evaluate the effect of two fat sources with divergent FA profiles on growth performance, carcass characteristics, pork quality, and pork anti-oxidant status of pigs grown to heavy slaughter weight (**SLW**) and its potential to be impacted by the VE supplementation level.

## Materials and Methods

This experiment was carried out in an environmentally controlled room at the University of Kentucky Swine Research Center. The animal slaughter and sample collection were performed at the University of Kentucky Meats Science Laboratory. The experiment was conducted under protocols approved by the Institutional Animal Care and Use Committee of the University of Kentucky.

### Animals, Experimental Design, and Housing

A total of 48 individually fed pigs (24 barrows, 24 gilts; 28.44 ± 2.69 kg) were selected from a pool of 120 pigs, and blocked by sire, body weight (**BW**), and sex, and then randomly assigned to one of the eight dietary treatments in a 2 × 4 factorial arrangement of fat sources and VE levels. Fat sources included TW and distiller’s corn oil (**DCO**). Vitamin E supplementation levels were four levels of α-tocopheryl acetate (**ATA**; 11, 40, 100, and 200 ppm). The form of VE was DL (all-rac)-ATA (ROVIMIX E 50 ADS, DSM Nutritional Products, Inc., Parsippany, NJ) in a dry form. As defined in NRC (2012), one IU VE is equal to 1 mg of DL-ATA. All pigs were housed in individual pens (0.61 × 2.44 m^2^) with free access to water and feed.

### Diets

The diets were corn-soybean meal based in mash form and fed to pigs with five feeding phases including 25 to 50 kg (phase 1), 50 to 75 kg (phase 2), 75 to 100 kg (phase 3), 100 to 125 kg (phase 4), and 125 to 150 kg (phase 5), respectively. All experimental diets were formulated to meet or exceed nutrient requirement estimates of NRC (2012) for growing-finishing pigs. Lysine levels for phase 4 were calculated with the formula provided by NRC (2012) because the pigs were slaughtered at ~150 kg which was greater than the final tabular weight category in NRC (2012). In phase 5 (125 to 150), lysine levels were chosen after consultation with industry nutritionists; the lysine level was higher in phase 5 of the current experiment than Wang et al. (2022a) that used the level calculated with the formula provided by NRC (2012). The fat inclusion level (5%) was based on the amount of DCO that might be realized from an aggressive use of distillers dried grains with solubles (**DDGS**; 45%) in the finishing diets. Formulas for each phase are provided in Table 1.

**Table 1.** Basal diet composition of diets with different fat source^1^ and VE level^2^ from phases 1 to 5 (as-fed basis)

Ingredient, %	Phase 1	Phase 2	Phase 3	Phase 4	Phase 5
Corn	62.85	69.55	73.81	77.04	80.17
Soybean meal, 48% CP	28.50	22.00	18.00	15.00	12.00
Fat (tallow or distiller’s corn oil)	5.00	5.00	5.00	5.00	5.00
L-Lysine HCl	0.22	0.24	0.21	0.17	0.22
DL-Methionine	0.12	0.09	0.04	0.01	0.01
L-Threonine	0.09	0.09	0.06	0.04	0.05
Limestone	1.08	0.99	0.88	0.77	0.68
Dicalcium phosphate	0.92	0.82	0.78	0.75	0.65
Salt	0.50	0.50	0.50	0.50	0.50
Vitamin premix^3^	0.02	0.02	0.02	0.02	0.02
Trace mineral premix^4^	0.15	0.15	0.15	0.15	0.15
Choline^5^	0.03	0.03	0.03	0.03	0.03
Santoquin^6^	0.02	0.02	0.02	0.02	0.02
AB-20^7^	0.50	0.50	0.50	0.50	0.50
Total	100.00	100.00	100.00	100.00	100.00
Calculated nutrient level, %					
ME, Mcal/kg	3.47	3.49	3.50	3.50	3.51
CP, %	19.13	16.58	14.94	13.70	12.59
SID Lys	1.04	0.90	0.78	0.67	0.64
SID Lys/ME	2.99	2.58	2.22	1.92	1.82
SID M + C	0.65	0.55	0.47	0.42	0.39
SID Thr	0.67	0.58	0.50	0.44	0.41
SID Trp	0.20	0.17	0.14	0.13	0.11
Ca	0.70	0.62	0.56	0.51	0.44
Total P	0.54	0.49	0.46	0.45	0.41
STTD P	0.32	0.29	0.27	0.26	0.23

^1^Fat treatments included distiller’s corn oil or tallow.

^2^Dietary VE treatments included four levels of α-tocopheryl-acetate (11, 40, 100, and 200 ppm) and were applied to each basal diet.

^3^Supplied the following per kg of diet: 7,000 IU of vitamin A; 1,500 IU of vitamin D_3_; 2.0 mg of vitamin K; 0.03 mg of vitamin B_12_; 7.0 mg of riboflavin; 25.0 mg of pantothenic acid; 20.0 mg of niacin; 1.0 mg of folic acid; 2.5 mg of vitamin B_6_; 2.0 mg of thiamin; and 0.15 mg of biotin.

^4^Supplied the following per kg of diet: 50 mg of Mn as manganese hydroxychloride; 100 mg of Fe as ferrous sulfate monohydrate; 125 mg of Zn as zinc hydroxychloride; 20 of Cu as tribasic copper chloride; 0.35 mg of I as Ca iodate; and 0.30 mg of Se as sodium selenite.

^5^Provided 150 mg of choline per kg of the final diet.

^6^Santoquin (Monsanto, St. Louis MO) supplied 130 mg/kg ethoxyquin to the final diet.

^7^Clay product from Prince Agri Products, Inc., Quincy IL.

Multiple batches of experimental diets were mixed for each phase. To minimize differences in nontreatment components of the diets, a basal diet for each of the two fat sources (TW and CO) was firstly mixed, to which the different VE treatments (11, 40, 100, and 200 ppm ATA) were then incorporated for each experiment, respectively. Representative samples of corn, soybean meal, and mixed feed were collected at the feed mill for every batch of experimental diets. Samples were stored at 4 °C until being composited and analyzed for concentration of ether extract (**EE**), crude protein (**CP**), calcium (**Ca**), and phosphorus (**P**) in each diet for verification of mixing accuracy.

### Growth, Harvest, and Carcass Characteristics

BW and feed disappearance were recorded every 2 wk to 108 kg and then weekly thereafter. Average daily gain (**ADG**), average daily feed intake (**ADFI**), and gain/feed ratio (**G:F**) were then calculated.

Pigs were slaughtered at about 150 kg live weight under USDA inspection. After being transferred to the meat lab, the pigs were slaughtered after a rest of at least 30 min. The slaughter process included electrical stunning, exsanguination, dehairing, evisceration, and carcass washing. During the process of slaughter, organs including liver, heart, kidney, spleen, and lungs were obtained and weighed. Then, 45 min pH of longissimus dorsi muscle (LM) at the 10th rib was measured in the meat cooler with an Accumet 50 pH meter (Fisher Scientific, Fairlawn, NJ, USA).

The hot carcass weight (**HCW**) and cold carcass weight (**CCW**), carcass length, backfat depth at four locations (1st rib, last rib, 10th rib and last lumbar), belly depth, longissimus dorsi muscle area (**LMA**), and 24 h pH were measured according to the methods described by McClelland et al. (2012). Briefly, HCW was recorded immediately after harvest to calculate dressing percentage [(HCW/BW) × 100)]. Shrink loss (%) was calculated using HCW and CCW {[1 – (CCW/ HCW)] × 100}. Following a 24-h chill (4 °C), carcass weight, backfat depth at the 10th rib, 1st rib, last rib, and last lumbar were measured. Carcass length was measured from the anterior edge of the symphysis pubic to the recess of the first rib. Belly depths were measured in six locations from the shoulder to flank end before the belly being boxed, and then frozen (−22 °C) until further analyses. The LMA and 24 h pH were measured from the left side of each carcass according to methods described by the National Pork Producers Council (NPPC, 2000). The Accumet 50 pH meter was used for the 24-h pH. After a 24-h cooler chill, primal cuts including Boston butt (IMPS #406), shoulder picnic (IMPS #405), loin (IMPS #412), and belly (IMPS #408; squared at each end) and spareribs were removed, and weighed individually according to Institutional Meat Purchasing Specifications (IMPS, 2010).

### Meat Quality

As soon as primal cut measurements were finished, slices of LM (~1.2 cm in thickness, and around 100 g) were obtained posterior to the 10th rib location. Drip loss was determined by suspending the sample, covered with a black plastic bag, from a hook in darkness and stored at 4 °C for 48 h. The samples were weighed before and after the process, drip loss percentage was determined by the equation:


$$\begin{array}{l} drip\;loss(48\;h,\%)=\frac{initial\;weight\;\text{\textasciitilde{}}--\;48\;h\;weight}{initial\;weight}\;\times \;100. \end{array}$$

About 10 cm section of the LM posterior to the 10th/11th rib interface were obtained and weighed prior to being vacuum packaged, boxed, and stored under refrigeration (4 °C) for 30 days to simulate the period of time between the packing plant and the retail grocery store. Loin samples were reweighed on days 7, 14, and 30 to determine purge loss at each stage to help determine when the majority of weight is lost during storage. Each time the samples were weighed, LM samples were taken out of the vacuum package, surface water removed with a paper towel, the weight recorded, and then the LM samples were vacuum packaged again.

Another 2.54 cm LM chop sample was cut from the LM between 7th and 8th rib. These LM samples were placed on foam trays and overwrapped in polyvinyl chloride (**PVC**) film. Subjective color, firmness, and marbling scores (NPPC, 2000) were evaluated by a trained experienced expert. The NPPC color scale (1 to 5) was used for color evaluation: 1 = pale pinkish to white; 5 dark purplish red. Similarly, the NPPC marbling scale (1 to 5, percentage fat in the LM), and NPPC firmness scale (1 to 5, 1 = very soft; 5 = very firm) was used. Afterwards, trays were then placed under fluorescent lighting at 4 °C to mimic retail conditions. Objective color measurements were made using a Hunter Lab Miniscan XE Plus colorimeter (Hunter Lab Associates, Reston, VA, USA) with the L*, a*, and b* scale at D65 light source, 2.54 cm diameter aperture, and 10° standard observer. The instrument was standardized before the analysis with black and white tiles that had been overwrapped with PVC film to adjust for the PVC cover upon the meat. Spectral reflectance was determined every 10 nm over the 400 to 700 nm range. Observations were made as soon as the fabrication of the carcass was finished and the meat color measurement was treated as the day 0 meat color. Afterwards, meat color was measured at 1100 hours each day for 1, 3, 5, and 7 d after the fabrication of the carcass; the color measurement was then analyzed for shelf life comparison among different dietary treatments. The a*/b* ratio, hue angle [tan^−1^(b*/a*)], and chroma $\left(\sqrt{a^{*2}}+b^{*2}\right)$ were calculated to show the development of color from red to yellow (Tapp et al., 2011).

### Oxidative Stability

After the meat color scores were taken on days 1, 3, 5, and 7, subsamples were immediately collected, flash frozen with liquid nitrogen, and stored at −80 °C for subsequent analysis of thiobarbituric acid reactive substances (**TBARS**) for oxidative stability.

Lipid oxidation was determined with the distillation method as described in Canto et al. (2016) to analyze TBARS. Briefly, a 5 g surface LM sample from each day at display was homogenized with 22.5 mL of 11% trichloroacetic acid (**TCA**) solution and filtered through Whatman no. 1 paper. Two milliliters of filtrate was mixed with 2 mL of aqueous solution of thiobarbituric acid (20 mM) and incubated at room temperature for 20 h. The absorbance values at 532 nm were then measured utilizing a UV-1800 spectrophotometer (Shimadzu Corporation, Kyoto, Japan). The value of the concentration of TBARS was calculated from a standard line based on the known concentration of standard malondialdehyde (Cayman, Ann Arbor, MI).

### Chemical Analyses of Fat and Diets

Samples of different fat sources were analyzed for FA profile, free FAs, moisture, insoluble, and unsaponifiables (**MIU**; NP Analytical Laboratories, St. Louis, MO). Fat was saponified with sodium hydroxide and methyl esters of the FAs were formed by reaction with boron trifluoride/methanol. FA methyl esters were then separated by gas chromatography (GC) and the GC peak area percent of each FA methyl ester were calculated as a percent of the total area of all FA methyl esters.

Diet samples were analyzed for EE as lipid, CP (or N × 6.25), Ca, and P for mixing verification. Dry matter was assessed according to the AOAC (1990) methods, involving overnight drying (105 °C) of the samples in a convection oven (Precision Scientific Co., Chicago, IL). EE was analyzed using Soxhlet extraction (920.39; AOAC 1990). Nitrogen was measured using Dumas methodology in an automatic nitrogen analyzer (Model FP 2000, LECO Corp., St. Joseph, MI). Around 4 g of feed samples were placed in an ash furnace at 550 °C for 3 h, and then dissolved in 40 mL of 1:3 hydrochloric acid/water on a hot plate that was preheated to 600 °C. The solution was quantitatively transferred to a 250-mL volumetric flask, brought to volume with deionized water and mixed thoroughly. Ca was assessed by flame atomic absorption spectrophotometry (Thermoelemental, SOLAAR M5; Thermo Electron Corp., Verona, WI) according to a modification of an AOAC (1990) procedure (method 975.03B). P was assessed by a gravimetric method (modification of method 968.08; AOAC, 1990). The VE concentrations in the diets and fat sources were determined with a normal phase HPLC system using fluorescence detection according to modification of an AOAC (1990) procedure (method 971.30). The concentration of tocopherols in fat sources was determined by the DSM research laboratory in Switzerland. Briefly, fat sample was mixed with milli-Q water and ethanol. Tocopherols were extracted from the aqueous suspension with hexane/BHT using a homogenizer. After centrifugation, an aliquot of the organic phase was injected onto a normal-phase HPLC system consisting of an autosampler, pump and fluorescence detector. A Lichrosorb Si 60 normal-phase column (250 mm × 4 mm i.d.; particle size, 5 μm) was used. The mobile phase was a solution of 4.5% dioxane in hexane. The retention times for α-, β-, γ-, and δ-tocopherol were determined, respectively, with purified tocopherols of different isoforms with > 95% purity from commercial sources. Quantification was performed by applying an external calibration.

### Statistical Analysis

Prior to analyses, all data were evaluated to identify any potential statistical outliers according to the test published by Barnett and Lewis (1994). Briefly, a set of data is ranked from low to high: X_L_, X_2_, ….X_H_, and the average and standard deviation are calculated, then suspected high or low outliers are tested by the following procedure: First, calculation of the statistic T: T = (X_H_ − Mean)/SD for a high value, or T = (X_L_ − Mean)/SD for the low value was made. Second, comparison of the value of T with the value from critical values for 95% confidence interval (under condition of this study, the critical value was 2.03) was made. When the calculated T was larger than the critical value for the measurement, then the X_L_ or X_H_ was a potential outlier at the level of 5% significance. Potential outliers were then evaluated by reviewing study notes for that animal as well as response measures and laboratory values for other animals on that particular dietary treatment to determine if the value would be excluded from the data set.

Data analyses were performed in SAS (SAS Inst. Inc., Gary, NC) by least squares analysis of variance using the generalized linear model (GLM) as a randomized complete block design. The individual pig served as the experimental unit. The model used was:


$$\begin{array}{l} Y\;=\;\mu \;+\;S_{l}+\;B_{i(l)}+\;V_{j}+\;F_{k}+\;VF_{jk}+\;SV_{lj}+\;SF_{lk}+n_{m\left(ijkl\right)}. \end{array}$$

In this equation, the parameters represent: *Y* = response variables, µ = overall population mean, *S*_l_ = sex (male or female), *B*_i(l)_ = block (i = 1, 2, 3….6), *V*_j_ = VE level (11, 40, 100, or 200 ppm), and *F*_k_ = fat sources (TW or DCO).

When interactions between main effects were significant, further least squares mean separations were accomplished using the PDIFF option of SAS to analyze the treatment effects. In addition, purge loss and shelf-life data were also analyzed as repeated measures to determine the response trends over time. Regression and contrast were also performed, if necessary, when interactions between time and main effect were observed.

Statistically significant differences were established at *P* ≤ 0.05, tendencies were established at *P* ≤ 0.10 for main effects. Sex effects were observed but are not presented or discussed because they were similar to that which has previously been published elsewhere. To focus attention on statistically relevant data in the tables, any *P*-value greater than 0.10 was replaced with “–”.

## Results

### Fat Quality and Analyzed Dietary Nutrient Levels

Apart from unexpected high free FA in the DCO (~10%), the FA profile of each fat source was as expected (Table 2). DCO had a higher content of UFAs especially PUFAs (53% vs. 2.75%). TW had a higher content of MUFA and SFA than DCO.

**Table 2.** Characteristics of different fat sources (as is basis)

Measurements^1^, %	Distiller’s corn oil	Tallow
Moisture	0.24	0.81
Free FA (calc. as Oleic Acid)	10.25	4.24
Unsaponifiable matter	1.90	0.33
Insolubles	<0.10	0.14
Trans FA	0.08	4.74
Total fat	97.30	96.70
FA profile, %		
C6:0 Caproic acid	<0.10	<0.10
C8:0 Caprylic acid	<0.10	<0.10
C10:0 Capric acid	<0.10	<0.10
C12:0 Lauric acid	<0.10	<0.10
C14:0 Myristic acid	<0.10	2.88
C16:0 Palmitic acid	12.73	24.30
C16:1n7 Palmitoleic acid	0.12	2.89
C17:0 Margaric acid	<0.10	1.32
C18:0 Stearic acid	2.11	19.85
C18:1n9T Elaidic acid	<0.10	4.68
C18:1n9C Oleic acid	26.30	34.25
C18:1n7C Vaccenic acid	0.99	1.44
C18:1 other cis isomers	<0.10	0.77
C19:0 Nonadecanoic acid	<0.10	0.20
C18:2 Other trans isomers	<0.10	0.31
C18:2n6 Linoleic acid	54.55	2.44
C20:0 Arachidic acid	0.38	<0.10
C18:3n6 Gamma linolenic acid	<0.10	<0.10
C20:1n9 cis Eicosenoic acid	<0.10	<0.10
C18:3n3 Linolenic acid	2.01	0.35
C20:2n6 Eicosadienoic acid	<0.10	<0.10
C20:3n6 Homo-Gamma-linolenic acid	0.15	<0.10
C20:5n3 Eicosapentaenoic acid	0.20	<0.10
Others	0.37	2.93
Saturated FAs	14.00	45.70
Monounsaturated FA	25.30	36.65
Polyunsaturated FA	53.00	2.75
Tocopherols, μg/mL		
α-tocopherol	136.04	58.93
β-tocopherol	LLQ	7.15
γ-tocopherol	481.29	365.00
δ-tocopherol	25.26	119.48
Total	642.59	550.56

^1^Values for the FA profile are the average of two samples.

LLQ, lower limit of quantification.

The analyzed nutrient levels are listed in Table 3. The fat from the common dietary ingredients, especially corn, made the total dietary fat content around 7%. Other than that, the nutrient levels were as expected.

**Table 3.** Analyzed nutrient levels^1^ of the basal diets from phases 1 to 5 (as-fed basis)

		Fat sources	
Phases	Measurements, %	Tallow	Distiller’s corn oil
Phase 1	Lipid	7.09	7.13
	CP	18.68	18.65
	Ca	0.80	0.79
	P	0.55	0.55
Phase 2	Lipid	7.70	7.17
	CP	16.42	16.35
	Ca	0.69	0.65
	P	0.51	0.50
Phase 3	Lipid	7.06	7.29
	CP	15.29	15.13
	Ca	0.62	0.65
	P	0.49	0.51
Phase 4	Lipid	7.21	7.04
	CP	13.53	13.65
	Ca	0.60	0.61
	P	0.47	0.48
Phase 5	Lipid	7.17	7.15
	CP	12.83	12.75
	Ca	0.53	0.52
	P	0.44	0.45

^1^Values are the average of four samples analyzed in duplicate.

### Growth Performance

No significant interaction between fat sources and dietary VE levels was detected on the growth performance (Table 4). Increasing dietary VE supplementation levels linearly increased ADG and ADFI in phase 1 (*P *< 0.05), phase 3 (*P *< 0.05), and phase 4 (*P *< 0.10; quadratic in ADFI, *P* = 0.06), but did not affect G:F. Regarding the fat source effect, the pigs fed the DCO diet tended to have a higher ADG in phase 4 (*P* = 0.07) and higher ADFI in phase 5 (*P* = 0.10) compared to the pigs fed the TW diet.

**Table 4.** Effect of different fat sources and VE supplementation levels on growth performance^1^ of pigs during each phase

Fat source	TW				DCO				SEM	*P*-value		
VE (ATA), ppm	11	40	100	200	11	40	100	200		VE^2^		Fat
										L	Q	
BW, kg												
Initial	28.27	29.03	28.58	28.27	27.82	27.90	28.88	28.80	0.439	–	–	–
Phase 1	53.22	51.18	53.30	52.69	51.26	51.33	52.09	54.73	0.944	0.03	–	–
Phase 2	73.33	74.01	75.22	72.95	74.46	73.10	75.07	74.46	0.887	–	–	–
Phase 3	98.43	99.06	99.03	101.38	98.28	97.67	98.43	100.02	1.092	0.02	–	–
Phase 4	123.38	123.74	122.55	123.07	123.60	123.68	124.96	125.42	1.021	–	–	0.10
Phase 5	146.28	148.23	147.49	144.92	149.16	148.78	148.02	151.12	1.846	–	–	–
ADG, kg/d												
Phase 1	1.13	1.01	1.12	1.11	1.07	1.07	1.05	1.18	0.033	0.04	–	–
Phase 2	1.15	1.11	1.18	1.10	1.07	1.12	1.12	1.22	0.049	–	–	–
Phase 3	1.08	1.09	1.15	1.12	1.03	1.06	1.11	1.22	0.039	<0.01	–	–
Phase 4	0.90	1.00	1.00	0.91	0.87	1.06	1.04	1.16	0.061	0.09	–	0.07
Phase 5	0.78	0.93	0.81	0.81	0.85	0.91	0.87	0.99	0.072	–	–	–
ADFI, kg/d												
Phase 1	2.15	2.08	2.20	2.13	2.05	2.07	2.12	2.32	0.070	0.03	–	–
Phase 2	2.55	2.49	2.71	2.45	2.47	2.51	2.60	2.75	0.104	–	–	–
Phase 3	2.79	2.67	2.98	2.74	2.68	2.73	2.84	3.06	0.089	0.02	–	–
Phase 4	2.66	2.94	3.07	2.78	2.74	2.94	3.01	3.18	0.126	0.10	0.06	–
Phase 5	2.80	2.94	2.84	2.68	2.97	3.05	3.04	3.20	0.158	–	–	0.10
G:F												
Phase 1	0.53	0.49	0.51	0.52	0.52	0.52	0.50	0.51	0.018	–	–	–
Phase 2	0.46	0.45	0.45	0.45	0.43	0.45	0.43	0.45	0.016	–	–	–
Phase 3	0.39	0.41	0.39	0.41	0.39	0.39	0.40	0.40	0.012	–	–	–
Phase 4	0.34	0.34	0.32	0.33	0.32	0.36	0.35	0.37	0.015	–	–	–
Phase 5	0.29	0.31	0.28	0.32	0.29	0.30	0.29	0.31	0.015	–	–	–

^1^Values are the average of six replicates. *P*-value greater than 0.10 was replaced with “–”.

^2^Linear (L) and quadratic (Q) responses based on dietary VE levels. No significant interaction was detected between levels of ATA and fat sources.

ATA, α-tocopheryl-acetate.

For the cumulative growth performance (Table 5), increasing dietary VE supplementation levels increased ADG in all cumulative phases from 28 to 150 kg (linear, *P* < 0.05 except for phases 1 and 2, *P* = 0.06). Interactions between fat sources and dietary VE supplementation levels were observed on cumulative ADG (*P* < 0.05) in phases 1 to 3 (28 to 100 kg) and phases 1 to 4 (28 to 125 kg) wherein the pigs fed the DCO diet, but not TW diet, had increased ADG with increasing dietary VE supplementation level (*P* < 0.05). A similar response was observed in ADFI in which a linear increase in ADFI with increasing VE supplementation levels from 11 to 200 ppm was observed in all cumulative phases (linear, *P* < 0.05 except for phases 1 to 2, *P* = 0.06, phases 1 to 4, linear and quadratic, *P* = 0.08). Interactions between fat sources and dietary VE supplementation levels were observed on cumulative ADFI (*P* < 0.05) in phases 1 to 3 (28 to 100 kg) and phases 1 to 4 (28 to 25 kg) wherein the pigs fed the DCO diet but not TW diet had increased ADFI with increasing dietary VE supplementation level (*P* < 0.05). Regarding the fat source effect, the pigs fed the DCO diet tended to have a higher ADFI for the overall phase (phases 1 to 5; *P* = 0.10) compared to the pigs fed the TW diet.

**Table 5.** Effect of different fat sources and VE supplementation levels on cumulative growth performance^1^ of pigs from 28 to 150 kg

Fat source	TW				DCO				SEM	*P*-value		
VE (ATA), ppm	11	40	100	200	11	40	100	200		VE^2^		Fat
										L	Q	
ADG, kg/d												
Phase 1	1.13	1.01	1.12	1.11	1.07	1.07	1.05	1.18	0.033	0.04	–	–
Phases 1–2	1.14	1.07	1.15	1.11	1.06	1.09	1.08	1.19	0.035	0.06	–	–
Phases 1–3	1.12	1.07	1.15	1.11	1.05	1.08	1.09	1.20	0.031	<0.01	–	–^I^
Phases 1–4	1.04	1.05	1.10	1.05	1.00	1.07	1.08	1.19	0.033	<0.01	–	–^I^
Phase s1–5	1.00	1.02	1.02	1.00	0.96	1.03	1.03	1.14	0.037	0.02	–	–
Average feed intake, kg/d												
Phase 1	2.15	2.08	2.20	2.13	2.05	2.07	2.12	2.32	0.070	0.03	–	–
Phases 1–2	2.33	2.28	2.42	2.28	2.25	2.28	2.35	2.50	0.072	0.06	–	–
Phases 1–3	2.50	2.40	2.61	2.45	2.40	2.44	2.51	2.70	0.062	<0.01	–	–^I^
Phases 1–4	2.51	2.55	2.73	2.53	2.50	2.58	2.65	2.82	0.060	<0.01	0.08	–^I^
Phases 1–5	2.62	2.63	2.75	2.58	2.60	2.68	2.73	2.91	0.058	<0.01	–	0.10
Gain/Feed												
Phase 1	0.53	0.49	0.51	0.52	0.52	0.52	0.50	0.51	0.018	–	–	–
Phases 1–2	0.49	0.47	0.48	0.49	0.47	0.48	0.46	0.48	0.015	–	–	–
Phases 1–3	0.45	0.45	0.45	0.45	0.44	0.44	0.44	0.45	0.012	–	–	–
Phases 1–4	0.41	0.41	0.41	0.42	0.40	0.42	0.41	0.42	0.010	–	–	–
Phases 1–5	0.38	0.39	0.37	0.39	0.37	0.38	0.38	0.39	0.009	–	–	–

^1^Values are the average of six replicates. *P*-value greater than 0.10 was replaced with “–”.

^2^Linear (L) and quadratic (Q) responses based on dietary VE levels. ^I^ Interaction between fat and dietary VE level, *P* < 0.05.

ATA, α-tocopheryl-acetate.

### Organ Size

Interactions between fat sources and dietary VE levels were not observed on absolute organ weight or relative organ weight (Table 6). The pigs fed the DCO diet had greater absolute liver and kidney weight (*P* < 0.05) with a tendency (*P* = 0.07) for greater relative liver weight than the pigs fed the TW diet. Quadratic responses were observed in absolute and relative weight of liver (*P* < 0.10) and lung (*P* < 0.05) wherein the absolute and relative weights decreased from 11 to 100 ppm of dietary VE supplementation level and increased afterward.

**Table 6.** Effect of different fat sources and VE supplementation levels on organ size^1^ of pigs

Fat source	TW				DCO				SEM	*P*-value		
VE (ATA), ppm	11	40	100	200	11	40	100	200		VE^2^		Fat
										L	Q	
Absolute organ weight, g												
Liver	1721.2	1435.6	1515.4	1713.4	1836.5	1841.3	1667.4	1825.7	126.06	–	0.08	0.03
Kidney	341.5	376.5	336.3	357.2	393.5	388.7	365.8	383.3	18.08	–	–	0.03
Heart	520.2	461.6	490.1	492.1	475.3	499.6	481.8	504.6	20.09	–	–	–
Lung	924.1	752.4	568.3	931.8	905.4	889.4	702.7	823.7	98.29	–	0.01	–
Spleen	168.0	157.8	177.9	174.1	181.6	182.1	167.5	177.1	11.71	–	–	–
Relative weight, % slaughter weight												
Liver	1.18	1.00	1.03	1.20	1.23	1.24	1.13	1.21	0.078	–	0.06	0.07
Kidney	0.23	0.26	0.23	0.25	0.27	0.26	0.25	0.25	0.014	–	–	–
Heart	0.36	0.32	0.33	0.35	0.32	0.34	0.33	0.33	0.017	–	–	–
Lung	0.63	0.51	0.39	0.65	0.61	0.60	0.47	0.54	0.065	–	<0.01	–
Spleen	0.12	0.11	0.12	0.12	0.12	0.12	0.11	0.12	0.008	–	–	–

^1^Values are the average of six replicates. *P*-value greater than 0.10 was replaced with “–”.

^2^Linear (L) and quadratic (Q) responses based on dietary VE levels. No significant interaction was detected between levels of ATA and fat sources.

ATA, α-tocopheryl-acetate.

### Primal Cut

Results of primal cut weights are provided in Table 7. With increasing dietary VE supplementation level, belly yield decreased in both absolute and relative weight (linear, *P* < 0.05). An effect of fat sources was only observed in the relative weight of loin (*P* < 0.05), wherein the pigs fed the TW diet had a greater relative weight than the pigs fed the DCO diet. An interaction between fat sources and dietary VE levels (*P* < 0.05) was observed in absolute weight of picnic shoulder wherein its weight increased in the pigs fed the DCO diet with increasing dietary VE supplementation levels but not in the pigs fed the TW diet.

**Table 7.** Effect of different fat sources and VE supplementation levels on primal cuts^1^ of pigs

Fat source	TW				DCO				SEM	*P*-value		
VE (ATA), ppm	11	40	100	200	11	40	100	200		VE^2^		Fat
										L	Q	
Primal cut, kg												
Boston butt	5.39	4.68	4.88	4.93	5.36	4.63	5.07	4.58	0.233	–	–	–
Picnic shoulder	5.11	5.60	5.44	4.83	4.93	5.51	5.08	5.73	0.240	–	–	–^I^
Loin	12.96	12.37	13.15	12.41	12.07	11.83	12.51	12.51	0.442	–	–	–
Spare rib	2.12	2.04	1.99	1.91	1.89	2.15	2.04	1.93	0.110	–	–	–
Ham	12.49	12.87	12.68	12.41	12.54	12.95	12.76	13.28	0.380	–	–	–
Belly	9.75	9.52	9.41	9.00	10.03	9.95	9.36	9.49	0.284	0.02	–	–
Primal cut, % live weight												
Boston butt	3.68	3.15	3.31	3.46	3.58	3.12	3.42	3.03	0.134	–	–	–
Picnic shoulder	3.49	3.78	3.69	3.41	3.28	3.73	3.43	3.79	0.152	–	–	–
Loin	8.88	8.35	8.92	8.74	8.08	7.98	8.45	8.28	0.302	–	–	0.03
Spare rib	1.45	1.38	1.35	1.35	1.26	1.45	1.38	1.28	0.080	–	–	–
Ham	8.54	8.69	8.60	8.71	8.39	8.75	8.62	8.79	0.238	–	–	–
Belly	6.66	6.43	6.38	6.32	6.70	6.71	6.32	6.28	0.161	0.02	–	–

^1^Values are average of six replicates. *P*-value greater than 0.10 was replaced with “–”.

^2^Linear (L) and quadratic (Q) responses based on dietary VE levels. ^I^ Interaction between fat and dietary VE level, *P* < 0.05.

ATA, α-tocopheryl-acetate.

### Carcass Traits

Interactions between fat sources and VE supplementation level on carcass traits (Table 8) were not detected except in 24 h pH that increased in the pigs fed the DCO diet with increasing dietary VE supplementation levels but decreased in the pigs fed the TW diet (*P* < 0.05). There were no significant differences in SLW, HCW, CCW, dressing percentage, shrink loss, carcass length, backfat depth, LM dimension, and LMA among different treatments. Increasing dietary VE supplementation levels from 11 to 200 ppm increased 45 min pH (quadratic, *P* < 0.01) and 24 h pH (quadratic, *P* = 0.01) with a peak at 40 to 100 ppm VE but did not affect the pH change over the 24-h chilling after slaughter. The pigs fed the TW diet had greater belly depth (*P* < 0.05) than the pigs fed the DCO diet.

**Table 8.** Effect of different fat sources and VE supplementation levels on carcass traits^1^ of pigs

Fat source	TW				DCO				SEM	*P*-value		
VE (ATA), ppm	11	40	100	200	11	40	100	200		VE^2^		Fat
										L	Q	
SLW, kg	146.2	148.2	147.5	142.4	149.7	148.2	148.0	151.1	2.68	–	–	0.08
HCW, kg	113.2	114.4	114.5	109.6	114.1	112.8	113.9	115.4	2.12	–	–	–
CCW, kg	110.5	111.6	111.1	106.8	111.4	109.5	111.4	112.8	2.18	–	–	–
Dressing, %	77.41	77.21	77.65	76.99	76.29	76.09	76.94	76.42	0.718	–	–	0.08
Shrink loss, %	2.33	2.48	2.96	2.60	2.36	2.88	2.23	2.29	0.608	–	–	–
45 min pH	5.90	6.13	6.15	5.91	5.82	6.19	6.10	6.10	0.086	–	<0.01	–
24 h pH	5.59	5.71	5.66	5.37	5.49	5.65	5.70	5.72	0.065	–	<0.01	–^I^
ΔpH	0.31	0.42	0.49	0.54	0.33	0.54	0.40	0.38	0.092	–	–	–
Carcass length, cm	83.57	82.80	83.71	83.82	82.42	85.25	83.82	84.24	1.189	–	–	–
Back fat depth, cm												
First rib	4.17	4.93	4.66	4.88	4.93	4.51	4.95	4.45	0.409	–	–	–
Last rib	3.35	3.71	3.43	3.00	3.33	3.40	3.43	3.70	0.295	–	–	–
10th rib	2.69	2.90	3.01	2.31	3.15	2.86	3.22	2.90	0.332	–	–	–
Last lumbar	2.34	2.72	2.12	1.73	2.29	2.48	2.46	2.41	0.284	–	–	–
Belly depth, cm	5.15	5.23	5.30	5.18	4.63	4.95	4.81	4.83	0.217	–	–	0.01
Loin muscle dimension^3^, cm												
Vertical	7.37	7.01	7.75	7.21	7.16	7.18	6.86	7.58	0.272	–	–	–
Horizontal	10.82	10.57	10.58	10.41	10.21	34.61	10.80	11.01	6.257	–	–	–
Area^4^, cm^2^	61.03	58.84	61.02	59.48	56.39	61.61	53.98	61.94	2.948	–	–	–

^1^Values are the average of six replicates. *P*-value greater than 0.10 was replaced with “–”.

^2^Linear (L) and quadratic (Q) responses based on dietary VE levels. ^I^Interaction between fat and dietary VE level, *P* < 0.05.

^3^Vertical distance refers to depth vertical to the 10th rib; Horizontal distance refers to width horizontal to the 10th ribs.

^4^Area was measured directly with a plastic standard grid as described by NPPC (2000).

ATA, α-tocopheryl-acetate; SLW, slaughter weight.

### Meat Quality and Shelf Life

The drip loss, purge loss, and subjective meat quality were not affected by dietary treatments (Table 9) but the purge loss increased over time (linear and quadratic, *P* < 0.01). Meat color, measured at a retail time of 1, 3, 5, and 7 d after the frozen LM samples were thawed and packaged in a retail foam tray overwrapped by PVC film, is presented in Table 10. Interactions between fat sources and dietary VE supplementation levels were observed in L* value (days 5 and 7, *P* < 0.05), a* value (day 7, *P* < 0.05), a*/b* (days 3 and 5, *P* < 0.05), and Hue angle (days 3 and 5, *P* < 0.05). The Chroma tended to increase with increasing dietary VE supplementation levels (*P *= 0.06) on day 7 whereas no effect was observed for fat source treatment. In further analysis of the color change, meat color differed with time (*P* < 0.05). Decreases in the L* value (quadratic, *P* < 0.01), a* value (linear and quadratic, *P* < 0.01), a*/b* (linear and quadratic, *P* < 0.01), and Chroma (linear, *P* < 0.01) were observed with increasing retail time while Hue angle and b* value increased (linear, *P* < 0.01) with time. No interaction between time and level of VE or fat sources were observed, which reflected the lack of effect of dietary VE on the developing of the color loss with time under the retail display.

**Table 10.** Effect of different fat sources and VE supplementation levels on shelf life of loin muscle measured as meat color^1^

Fat source	TW				DCO				SEM	*P*-value		
VE (ATA), ppm	11	40	100	200	11	40	100	200		VE^2^		Fat
										L	Q	
L*^3^												
Day 1	53.40	48.32	56.98	51.86	51.51	51.08	51.71	54.43	2.083	–	–	–
Day 3	56.23	50.58	57.66	53.23	52.27	52.68	53.06	55.44	2.014	–	–	–
Day 5	56.05	51.24	58.25	54.19	53.99	53.91	53.58	57.28	2.051	–	–	–^I^
Day 7	53.02	48.93	56.80	50.29	52.63	50.29	49.50	54.28	2.205	–	–	–^I^
a*^4^												
Day 1	7.52	8.26	6.80	7.52	7.54	7.37	7.53	7.52	0.659	–	–	–
Day 3	7.84	9.44	7.46	8.54	8.36	8.07	8.37	7.48	0.663	–	–	–
Day 5	6.05	6.88	6.29	7.26	6.28	6.46	7.34	5.71	0.599	–	–	–
Day 7	6.53	6.50	5.47	7.56	5.84	6.34	6.90	5.99	0.641	–	–	–^I^
b*^5^												
Day 1	14.88	13.51	15.11	14.44	13.93	13.75	14.19	14.78	0.470	–	–	–
Day 3	16.09	15.31	16.35	15.76	15.49	15.38	15.76	15.74	0.354	–	–	–
Day 5	15.86	15.01	15.83	15.33	15.00	14.92	15.49	15.62	0.360	–	–	–
Day 7	17.13	15.21	17.10	16.81	16.09	16.54	17.47	17.57	0.658	–	–	–
a*/b*^6^												
Day 1	0.51	0.62	0.45	0.52	0.54	0.54	0.53	0.51	0.046	–	–	–
Day 3	0.49	0.62	0.46	0.54	0.54	0.52	0.53	0.48	0.041	–	–	–^I^
Day 5	0.38	0.46	0.40	0.47	0.42	0.43	0.47	0.37	0.043	–	–	–^I^
Day 7	0.39	0.44	0.32	0.45	0.36	0.39	0.40	0.35	0.048	–	–	–
Hue angle^7^												
Day 1	1.10	1.02	1.15	1.10	1.07	1.08	1.08	1.10	0.033	–	–	–
Day 3	1.12	1.02	1.14	1.08	1.08	1.09	1.08	1.13	0.031	–	–	–^I^
Day 5	1.21	1.15	1.19	1.13	1.18	1.17	1.13	1.22	0.033	–	–	–^I^
Day 7	1.21	1.16	1.26	1.15	1.22	1.20	1.19	1.24	0.039	–	–	–
Chroma^8^												
Day 1	16.69	15.85	16.58	16.31	15.85	15.62	16.07	16.65	0.587	–	–	–
Day 3	17.96	18.01	18.01	17.92	17.62	17.38	17.85	17.46	0.503	–	–	–
Day 5	16.99	16.56	17.05	16.97	16.28	16.27	17.15	16.65	0.422	–	–	–
Day 7	18.43	16.58	17.98	18.53	17.14	17.72	18.80	18.62	0.621	0.06	–	–

^1^Values are average of six replicates. *P*-value greater than 0.10 was replaced with “–”.

^2^Linear (L) and quadratic (Q) responses based on dietary VE levels. ^I^ Interaction between fat and dietary VE level, *P* < 0.05.

^3^Time effect, quadratic, *P* < 0.01; interaction between time and fat sources, *P* = 0.10. No interaction between time and dietary VE levels was observed.

^4^Time effect, linear and quadratic, *P* < 0.01. No interaction between time and fat sources or dietary VE levels was observed.

^5^Time effect, linear, *P* < 0.01. No interaction between time and fat sources or dietary VE levels was observed.

^6^Time effect, linear and quadratic, *P* < 0.01. No interaction between time and fat sources or dietary VE levels was observed.

^7^Time effect, linear and quadratic, *P* < 0.01. No interaction between time and fat sources or dietary VE levels was observed.

^8^Time effect, linear, *P* < 0.01. No interaction between time and fat sources or dietary VE levels was observed.

ATA, α-tocopheryl-acetate.

**Table 9.** Effect of different fat sources and VE supplementation levels on meat quality^1^ of pigs

Fat source:	TW				DCO				SEM	*P*-value		
VE (ATA), ppm:	11	40	100	200	11	40	100	200		VE^2^		Fat
										L	Q	
Drip loss, %	5.99	5.63	5.67	5.21	7.38	5.20	6.08	5.36	0.789	–	–	–
Purge loss^3^, %												
Day 7	3.60	4.50	4.12	5.42	4.65	3.88	4.34	4.51	0.877	–	–	–
Day 14	8.13	9.14	8.94	8.90	9.22	9.73	9.43	9.35	1.201	–	–	–
Day 30	12.02	12.89	12.70	11.71	11.42	13.54	12.00	13.82	1.419	–	–	–
Subjective meat quality^4^												
Color	2.60	3.20	2.67	2.40	3.00	3.00	2.50	2.83	0.327	–	–	–
Marbling	1.40	2.00	2.00	2.20	1.80	2.00	2.17	1.33	0.366	–	–	–
Firmness	2.40	2.80	2.50	2.20	2.60	2.75	2.17	1.83	0.363	–	–	–

^1^Values are average of six replicates.*P*-value greater than 0.10 was replaced with “–”.

^2^Linear (L) and quadratic (Q) responses based on dietary VE levels. No significant interaction was detected between levels of ATA and fat sources.

^3^Time effect, linear and quadratic, *P* < 0.01; no interaction between time and fat sources or dietary VE treatments was observed.

^4^Color: National pork producers council (NPPC) color scale (1–5): 1 = pale pinkish to white; 5 = dark purplish red. Marbling: NPPC marbling scale (1–5): percentage fat in the loin muscle. Firmness: NPPC firmness scale (1–5): 1 = very soft; 5 = very firm.

ATA, α-tocopheryl-acetate.

The TBARS values were measured to assess oxidative stability (Table 11). No interactions between fat sources and dietary VE supplementation levels were observed in this measurement. Dietary VE levels did not affect TBARS values from days 1 to 5 after slaughter; however, on day 7 increasing dietary VE supplementation level decreased TBARS values (linear, *P* < 0.01). Regarding the fat source effect, the pigs fed the DCO diet had greater TBARS value than the pigs fed the TW diet from days 1 to 7 (*P* < 0.05; day 5, *P *= 0.09). The TBARS value in the LM increased with time (linear and quadratic, *P* < 0.01), and interactions were observed between time and fat sources and dietary VE levels (*P* < 0.05). Further comparison of the slopes for the development of TBARS along with time indicated that the TBARS in the LM from pigs fed 11 ppm increased with a greater (*P* < 0.05) slope compared to that from pigs fed 40, 100, and 200 ppm VE and the TBARS content from pigs fed the DCO diet increased with a greater (*P* < 0.05) slope compared to those from the pigs fed the TW diets.

**Table 11.** Effect of different fat sources and VE supplementation levels on shelf life of loin muscle measured as TBARS^1^ (µg MDA/kg wet meat)

Fat source	TW				DCO				SEM	*P*-value		
VE (ATA), ppm	11	40	100	200	11	40	100	200		VE^2^		Fat
										L	Q	
Day 1^3^	0.276	0.231	0.218	0.218	0.259	0.289	0.292	0.293	0.026	–	–	0.02
Day 3	0.320	0.313	0.293	0.300	0.367	0.334	0.361	0.340	0.016	–	–	<0.01
Day 5	0.414	0.354	0.327	0.388	0.435	0.395	0.429	0.374	0.030	–	–	0.09
Day 7	0.735	0.585	0.534	0.562	0.871	0.749	0.783	0.653	0.052	<0.01	–	<0.01

^1^Values are average of six replicates. *P*-value greater than 0.10 was replaced with “–”.

^2^Linear (L) and quadratic (Q) responses based on dietary VE levels. No interaction between fat sources and dietary VE levels was observed.

^3^Time effect, linear and quadratic, *P* < 0.0001, with interaction with both fat sources and levels of VE (*P* < 0.05).

ATA, α-tocopheryl-acetate.

## Discussion

The FA profile of different fat sources was close to values listed in NRC (2012) and the MIU content of the two fats were both less than 2.5%. The results confirmed their representativeness as designed fat sources. The FA composition and other fat-related characteristics were similar with Wang et al. (2022a, 2022b) as the same sources were used in both studies.

Although there were no notably significant effects of fat sources on growth performance, the pigs fed the DCO diet tended to have greater ADG in phase 4 and ADFI in phase 5 and overall period compared to the pigs fed the TW diet. This result agrees with our previous study (Wang et al., 2022a) that reported that the pigs fed the DCO diet had greater growth rate and feed intake than the pigs fed the TW diet in late finishing periods. The current study used a mean value of ME for TW and DCO provided in the NRC (2012) for the diet formulation but DCO actually has a greater ME value than TW (NRC, 2012). With the slightly greater feed intake, the pigs fed the DCO diet presumably consumed a greater amount of energy. With a potential increase in energy digestibility with the DCO diet due to its high PUFA content (Powles et al., 1994), these factors may explain the greater growth rate in pigs fed the DCO diet than the TW diet. In addition, even though energy density in the DCO diet might be greater than the TW diet, voluntary feed intake of pigs was not reduced, which suggests that the DCO diet may potentially have had better palatability than the TW diet. Davis et al. (2015) reported that pigs fed diets with 5% TW had lower feed intake compared with the pigs fed the diets with no fat or 30% DDGS. However, this result is in contrast to Liu et al. (2018) who reported that 6% TW in the finishing pig diet had slightly greater feed intake than soybean oil but no difference in overall growth rate.

In the current study, increasing dietary VE supplementation from 11 to 200 ppm linearly increased ADG and ADFI during phases 1, 3, and 4, and resulted in a linear increase in cumulative ADG and ADFI including the overall period. This result is in agreement with several previous studies (Asghar et al., 1991; Niculita et al., 2007). Asghar et al. (1991) reported that growth rate and feed efficiency increased significantly by supplementing diets with 100 and 200 ppm of VE compared to the diet containing 10 ppm of VE. It has been also reported that finishing pigs fed diets containing 300 ppm of VE had greater growth rate than those fed diets with 11 ppm of VE (Niculita et al., 2007). This linear increase in growth rate and feed intake indicates that the VE requirement estimate for pigs suggested by NRC (2012) requires adjustment. Similar to our previous study (Wang et al., 2022a), the response of pigs to dietary VE supplementation level was mainly observed in pigs over 75 kg with an interaction between fat source and dietary VE supplementation levels in the cumulative growth rate and feed intake in phases 1 to 3 and 1 to 4 wherein the pigs fed the DCO diet, but not the TW diet, had increased ADG with increased dietary VE. This interaction may indicate a situation-dependent need for VE supplementation level. However, there are also several publications that did not detect this effect of dietary VE on growth performance (Cannon et al., 1996; Lauridsen et al.,1999; Onibi et al., 2000). Lauridsen et al. (1999) reported no beneficial effect of supplementation of high levels of VE in the swine diets in which feeding pigs with 200 ppm dietary VE reduced ADG compared to pigs fed either 0 or 100 ppm dietary VE. Onibi et al. (2000) also reported no beneficial effect of dietary VE supplementation level at 200 ppm on growth performance compared to an unsupplemented control group. This contrary result may be due to lighter slaughter weight (110 to 111 kg) used in these previous studies. Therefore, further studies are needed to demonstrate the effects of dietary VE supplementation levels on growth performance of pigs with heavier final weight and/or a longer duration of feeding diets with high VE level.

Regarding the results of organ size, although there were quadratic responses in absolute and relative weight of liver and lung with the lowest value at 100 ppm VE level with increasing dietary VE levels (which may indirectly suggest more efficient organ function), those did not differ between 11 and 200 ppm VE levels. In contrast, Wang et al. (2022a) reported that absolute and relative liver weight increased when 200 ppm of VE was supplemented to the diets. There is no clear explanation on this response, but the 200 ppm of VE is not considered as a toxic level to swine (Pinelli-Saavedra, 2003).

In the current study, there was no effect of increasing VE supplementation level on primal cuts and carcass characteristics, and meat quality including meat color with exceptions in a few measurements. Cannon et al. (1996) and Boler et al. (2009) also reported no effect of VE supplementation level on carcass traits including backfat thickness, carcass weight, and dressing percentage. However, Corino et al. (1999) reported that HCW and dressing percentage increased with increasing dietary VE supplementation level. Absolute and relative belly weight decreased linearly with increasing VE supplementation level in the current study. Similarly, Wang et al. (2022a) reported that belly depth decreased when 200 ppm of VE was supplemented to the diet although there was no difference in total weight of that primal cut. In addition, pork pH at 45 min and 24 h postmortem increased quadratically with increasing VE supplementation level with the highest value in pigs fed the 40 and 100 ppm of VE levels. This result agrees with Wang et al. (2022a) who reported that 200 ppm of VE supplementation slightly increased 45 min pH of pork. As low 45min pH of pork indicates a potential incidence of PSE (pale, soft, and exudative) meat (Kim et al., 2015), over 40 ppm of VE supplementation to the swine diets could reduce the possibility of inducing PSE pork at slaughter.

In organ weight and carcass traits, there were no significant differences by different fat sources except that the pigs fed the DCO diet had greater liver and kidney weight but lower relative loin weight, belly depth and dressing percentage than the pigs fed the TW diet. Kyle et al. (2014) reported a negative correlation between belly thickness and PUFA content and IV, and the DCO generally contains greater PUFA content with higher IV than the TW and it has been suggested that inclusion of DDGS in the swine diet could reduce dressing percentage (Stein and Shurson, 2009) although there is, at yet, no clear explanation for this response. Cliplef and McKay (1993) reported that pigs with reduced backfat thickness and increased growth rate had increased weight of visceral organs and decreased carcass weight and dressing percentage. Therefore, this increase in organ weight might result in lower dressing percentage in the DCO treatment than the TW treatment in the current study. However, several previous studies reported no effect of dietary fat source and FA composition in carcass traits and organ weight (Smith et al., 1999; Lee et al., 2013; Stephenson et al., 2016). Therefore, further studies are necessary to clearly demonstrate the fat source effect on organ size, carcass characteristics, and primal cuts as there were no notable differences in growth rate, backfat thickness, and dressing percentage between the two dietary fat sources in the current study.

There were no effects of increasing dietary VE supplementation level or dietary fat sources in drip loss, purge loss, marbling, and firmness. When shelf life was measured in terms of meat color, no effect of dietary VE supplementation or dietary fat sources were detected on the meat color development when analyzed as comparisons between slopes of the linear trend of L*, a*, b*, a*/b*, Hue, and Chroma along with time. Although scattered interaction between fat sources and dietary VE levels were detected when analyzed daily, no clear trend was observed. This result is in agreement with previous studies on pork color in response to dietary VE supplementation (Ohene-Adjei et al., 2004; Guo et al., 2006). A consistent effect of dietary VE supplementation on meat color has been extensively reported in ruminant animals including cattle, goat, and lamb (Rentfrow et al., 2004; Suman et al., 2007; Jose et al., 2018; Possamai et al., 2018), but this effect is inconsistent in pork. The inconsistent result might be due to the structural differences of myoglobin (Mb) from pigs and cattle. When Mb was incubated with the 4-hydroxy-2-nonenal (**HNE**), a product of lipid oxidation, only mono-adducts of HNE with porcine Mb were detected with three histidine residues (HIS 24, 36, and 119) in porcine Mb that were readily adducted by HNE, whereas in bovine Mb seven histidine residues (HIS 24, 36, 81, 88, 93, 119, and 152) were adducted (Suman et al., 2007). The Mb in pork is less affected by the lipid oxidation occurring compared to beef.

In TBARS values, the pork from the DCO treatment pigs had greater TBARS values than that from the TW treatment pigs for 7 d postmortem. Wang et al. (2022b) reported greater PUFA content and IV in backfat, belly fat and liver when pigs were fed a 5% DCO diet compared with a 5% TW diet. Monahan et al. (1990) also reported that muscle on day 4 of storage from pigs fed the TW diet from 7 to 84 kg BW were less susceptible to iron-induced lipid oxidation compared with those fed the soy oil diet. High PUFA content in the pork could increase susceptibility of pork to lipid peroxidation and rancidity (Monahan et al., 1990; Mitchaothai et al., 2007; Browne et al., 2013). Another possible reason for increased TBARS values in the DCO treatment compared with the TW treatment in the present study were clearly lower alpha-tocopherol deposition in the muscle of pigs fed the DCO treatment compared to the TW treatment (Wang et al., 2023 [in press]. Therefore, high PUFA deposition in pigs fed the DCO diet could increase the susceptibility to lipid peroxidation in muscle resulting in reduced shelf-life of pork.

The TBARS values at day 7 postmortem were improved (i.e., decreased linearly) with increasing dietary VE supplementation level. Corino et al. (1999) reported that muscle samples on day 6 of storage had significantly reduced TBARS values when 100 and 300 IU/kg VE was supplemented to the diets for pigs from 120 to 160 kg BW compared with the control diet supplemented with 25 mg VE/kg and there was a positive correlation between muscle alpha-tocopherol and TBARS between plasma alpha-tocopherol and TBARSon day 6 of storage. Monahan et al. (1990) also reported that TBARS values in the muscle from pigs fed the 200 ppm VE diet on day 4 of storage were significantly lower than those from the pigs fed the 10 to 50 ppm VE diet regardless of fat sources (TW or soy oil). This result confirms that increasing vitamin E improves shelf-life of pork and stability to lipid peroxidation of pork.

In conclusion, increasing vitamin VE supplementation level enhances growth rate and feed intake in late finishing period and reduces lipid peroxidation of pork. Further, diets containing high levels of DCO as a fat source can negatively affect pork shelf-life and carcass characteristics compared to diets with TW.
